# Pituitary-Specific Overexpression of Porcine Follicle-Stimulating Hormone Leads to Improvement of Female Fecundity in BAC Transgenic Mice

**DOI:** 10.1371/journal.pone.0042335

**Published:** 2012-07-31

**Authors:** Mingjun Bi, Jia Tong, Fei Chang, Jing Wang, Hengxi Wei, Yunping Dai, Mingxing Chu, Yaofeng Zhao, Ning Li

**Affiliations:** 1 State Key Laboratory for Agrobiotechnology, College of Biological Sciences, China Agricultural University, Beijing, China; 2 Guangdong Provincial Key Laboratory of Agro-Animal Genomics and Molecular Breeding, College of Animal Sciences, South China Agricultural University, Guangzhou, China; 3 Key Laboratory of Farm Animal Genetic Resources and Germplasm Innovation of Ministry of Agriculture Institute of Animal Science, Chinese Academy of Agricultural Sciences, Beijing, China; University of Munich, Germany

## Abstract

Follicle-stimulating hormone (FSH) is a pituitary glycoprotein that, together with luteinizing hormone, plays a crucial role in ovarian folliculogenesis and female fertility. We previously found that FSH beta is a major gene controlling high prolificacy of Chinese Erhualian pigs. To directly study the biological effects on reproductive function of porcine FSH (pFSH) for polyovulatory species, we generated a novel gain-of-function mouse model using a bacterial artificial chromosome (BAC) system to jointly introduce 92 kb and 165 kb genomic fragments comprising the pFSH α- and β-subunit genes. These directed the physiological expression of pFSH with the same temporal and spatial pattern as endogenous FSH in female transgenic (TG) mice. Serum levels of biologically active pFSH heterodimers in independent TG lines ranged from 6.36 to 19.83 IU/L. High basal pFSH activity led to a significant reduction of serum LH and testosterone levels in TG females compared to wild-type (WT) littermates, yet endogenous FSH and estradiol levels were significantly elevated. Interestingly, ovarian histology showed that the number of corpora lutea was significantly higher at 14 and 28 weeks of age in TG females and breeding curves revealed that mean litter sizes of TG females were obviously larger than for WT littermates before 52 weeks of age. These findings indicate that pituitary-specific overexpression of pFSH within physiological boundaries can increase ovulation rate and litter size, but it does not cause reproductive defects. Therefore, our TG mouse model provides exciting insights for investigating the actions of pFSH in vivo.

## Introduction

The glycoprotein hormone superfamily includes follicle-stimulating hormone (FSH), luteinizing hormone (LH), and thyroid-stimulating hormone (TSH), as well as placental chorionic gonadotropin (CG) which is present only in primates and horse. These glycoprotein members contain a common α subunit which is noncovalently linked to a hormone-specific β subunit to form biologically active heterodimers [1]. The glycoprotein α subunit and the hormone-specific FSH β subunit are expressed in gonadotroph cells of the anterior pituitary and are regulated by the pulsatile release of hypothalamic gonadotropin-releasing hormone (GnRH) and by gonadal steroids and peptides, activins, and inhibins [2], [3], [4]. In females, FSH binds to its cognate receptor on ovarian granulosa cells to control the maturation of follicles. FSH plays a major role in antral follicle development and, together with LH, stimulates preovulatory follicular growth, although primordial follicle development to the preantral stage may proceed independently of FSH [5], [6].

Transgenic (TG) mice harboring the human FSH two subunit genes have been used previously to explore the role of FSH in reproductive function, and these animals have provided important information about the pathological effects of FSH levels that exceed normal physiological boundaries [7], [8], [9]. These models are useful for studying human pathologies and for mimicking reproductive diseases. Human FSH is, however, ectopically expressed in these models and is not regulated by the hypothalamic–pituitary–gonadal axis; therefore, the models do not reproduce the chromosomal environment of the human FSH gene. In the early 1990s, Kumar and colleagues first generated TG mice harboring a 10 kb human FSH β-subunit gene derived from cosmid clones [10]. The human FSH β-subunit gene was expressed at a high basal level in the pituitary of the male TG mice and demonstrated steroid hormone regulation [11], [12]; however, the 10 kb genomic DNA clone used to generate the TG mice lacked distant regulatory elements and did not adequately keep expression within normal physiological range without natural homeostatic control mechanisms because the correct spatiotemporal expression of a gene is often controlled by appropriate promoter and long-range *cis*-regulatory elements in the global chromatin structure [13]. Our laboratory previously verified FSH beta is a major gene controlling high prolificacy of Chinese Erhualian pig that is the most prolific pig breed of the world [14], [15]. Therefore, to evaluate the physiological effects of normal expression profile of porcine FSH (pFSH) for polyovulatory species in vivo, we took advantage of large-fragment TG strategies to establish TG mice.

The yeast artificial chromosome (YAC) system has been applied widely in large-fragment TG research, but as a vector system YACs are difficult to purify and often result in rearrangements, and this restricts their applicability [16]. One approach to solve this problem is to introduce the gene of interest using a bacterial artificial chromosome (BAC) system. Based on the *Escherichia coli* F factor, the BAC system comprises a single-copy circular DNA molecule inserted into a bacterial cell. BACs are more stable and easier to purify than cosmids or YACs. The average insertion using a BAC vector is ∼150 kb, a size compatible with transgene inserts containing long-range *cis*-regulatory elements, and with minimal chromosomal position effects on expression than smaller transgenes [17], [18].

Here, we screened a porcine BAC library and generated TG mice bearing selected BAC clones, including the complete pFSH α- and β-subunit genes. We used these mice to investigate the effects of pFSH on gonadal development and function in a gain-of-function mouse model for polytocous animals.

## Materials and Methods

### Porcine BAC library screening

BAC clones were isolated from a porcine BAC library (constructed with genomic DNA from a male Erhualian pig by the State Key Laboratory for Agrobiotechnology) [19] by three-dimensional PCR-based screening. BAC DNA constructed in pBeloBAC11 was purified using the Large-Construct Kit (QIAGEN, Germany). Insert sizes were confirmed by pulsed-field gel electrophoresis (PFGE) of *Not*I-digested BAC DNA on a 1% agarose gel (CHEF-DR III; Bio-Rad, Hercules, CA, USA) with the MidRange II PFG marker (New England BioLabs). To identify the BAC clones, the complete BAC DNA inserts were sequenced (Majorbio, Shanghai, China) and compared with the published gene sequences.

### Preparation of BAC DNA and generation of BAC transgenic mice

To prepare DNA for microinjection, *Not*I-digested fragments were size-fractionated for 14 h using PFGE on a 1% low-melting-point agarose gel (6 V/cm; angle, 120°; switching time, 0.1–40 s). The linearized fragments (devoid of vector sequences and without exposure to UV light) were electroeluted as described [20], [21] and purified overnight by dialysis against microinjection buffer (5 mM Tris-HCl, pH 7.4; 5 mM NaCl; 0.1 mM EDTA) by floating on a Millipore VSWP 2500 filter disc (0.05 µm pore size). Two fragments (adjusted to 1 ng/µl each) were co-injected into mouse zygotes (KM strain; Beijing Laboratory Animal Research Centre) according to standard procedures [22]. Stable pedigrees of TG mice were obtained by crossing double transgene-positive founder mice to control wild-type (WT) littermates. Mice were housed under controlled temperature (22±2°C), humidity (40–60%) and lighting (12 h light/12 h darkness) with food and water ad libitum. All animal studies were approved by the Animal Welfare Committee of China Agricultural University with approval number SKLAB-2011-01-02.

### TG examination by PCR and Southern blot

TG founders and offspring were identified by PCR analysis of genomic DNA derived from tail biopsies. To evaluate the integrity of the integrated DNA, 5′-and 3′-flanking sequences of the BAC inserts were PCR amplified separately from TG mouse DNA. PCR primers are shown in Table S1.

TG founder mice were confirmed by Southern blot analysis. Tail DNA samples (10 µg) were digested with *Bam*HI and electrophoresed through a 1% agarose gel followed by transfer to positively charged nylon membrane (Roche Applied Science, Mannheim, Germany). The membrane was hybridized with a digoxigenin (DIG)-labeled probe (998 bp for *pFSHα* and 1008 bp for *pFSHβ*) generated using the Roche DIG-PCR DNA Labeling Kit, with specific *pFSHα* or *pFSHβ* subfragments as the template; hybridization signals were detected using Roche DIG DNA Labeling and Detection Kit according to the manufacturer's instructions. Transgene copy number was estimated by comparing the hybridization signal density using Quantity One 4.5 software (Bio-Rad).

### RT-PCR

Total RNA was prepared from multiple tissues using TRIzol Reagent (Tiangen, Beijing, China) followed by DNase I digestion, as described [23]. RNA (2 µg) was reverse transcribed into first-strand cDNA using oligo(dT) primers (Promega, Madison, WI, USA). PCR with gene-specific primers was then used to detect transgene expression [23]. The murine housekeeping gene glyceraldehyde-3-phosphate dehydrogenase (*Gapdh*, NC_000072.6) was used as an internal control. Special primer sequences for each product were as follows: *pFSHα* (5′-GGGTGCCCCAATCTATCAGT-3′, 5′-GGCATTCGGTGTGGTTCTCC-3′), *pFSHβ* (5′-AAGCCATCTGCTGCAATAGCT-3′, 5′-GGTGAGCACAGCCAGGTACTT-3′), *Gapdh* (5′-AGGCCGGTGCTGAGTATGTC-3′, 5′-TGCCTGCTTCACCACCTTCT-3′). Amplified fragments were separated on a 1.5% agarose gel and visualized by ethidium bromide staining.

### Quantitative real-time PCR

All of quantitative real-time PCR analyses were performed using a LightCycler 480 II system (Roche) and accompanying software according to the manufacturer's instructions. PCR products were quantified by measuring the fluorescent dye SYBR green (Roche) bound to double-stranded DNA. A standard PCR protocol was used for all primers (Table S1): 95°C for 10 min; 40 cycles of 95°C for 10 s, 60°C for 15 s and 72°C for 10 s; followed by 95°C for 5 s, 65°C for 1 min, and 97°C continuous to generate the melting curve. Levels of gene mRNA were standardized to levels of mouse β-actin (internal control, NC_000071.6) mRNA in each sample and the relative fold change was calculated by the 2^−ΔΔCt^ method [24]. Copy number of a given target sequence was quantified by standard curve method as described previously [25], [26]. The standard curve was established by a standard set of mixtures representing 1, 2, 4, 8 and 16 copies of plasmid DNA in 6 ng of WT mouse genomic DNA. The conserved mouse housekeeping gene fatty acid binding protein (*Fabpi*, NC_000069.6) served as an internal control to calculate transgene copy number. Data were collected from three independent experiments.

### Northern blot analysis

Total RNA was extracted as described [23] and dissolved in formamide. Aliquots (20 µg) were denatured and separated on a 1.2% agarose–formaldehyde gel. Fragmented RNA was transferred to a positively charged nylon membrane (Roche) and cross-linked to the membrane by UV irradiation using a GS Gene Linker UV Chamber (Bio-Rad). The membrane was then hybridized in turn with different DIG-labeled probes (a 316 bp cDNA for *pFSHα*, a 347 bp cDNA for *pFSHβ*, and a 939 bp cDNA for *Gapdh* as an internal control) generated using the DIG High Prime DNA Labeling Kit (Roche). Blots were stripped after each hybridization and re-hybridized with the next probe; hybridization signals were detected using the DIG DNA Labeling and Detection Kit (Roche).

### Histological analysis

Tissues were collected, fixed for ∼5 days in 4% formalin solution, embedded in paraffin, and serially sectioned (5 µm). After deparaffinization, tissue sections were stained with hematoxylin and eosin (H&E) and examined by light microscope (Nikon, Japan) using brightfield optics as described previously [5], [27]. Total numbers of ovarian corpora lutea were determined by morphology using aligned, captured (four-objective) microscope images taken every tenth ovarian section [28]. All sections were examined without knowledge of the genotype of the mice.

### Hormone assays

Blood was collected by retro-orbital puncture from TG mice and WT littermates at 19–21 weeks of age and clotted at 4°C overnight. Serum samples were separated by centrifugation at 3500 rpm for 10 min and stored at −80°C until further analysis. Serum levels of TG pFSH were measured by ELISA using a polyclonal antibody (Thermo, Rockford, IL, USA) specific to porcine FSH. Serum levels of endogenous mouse FSH, LH, estradiol, and testosterone were measured by RIA (Blue Gene, Shanghai, China) according to the manufacturer's instructions. All samples were tested in triplicate. The sensitivities of the mouse FSH, LH, estradiol, and testosterone assays were 0.4 IU/L, 0.3 IU/L, 0.5 pg/ml, and 0.002 ng/ml, respectively. The inter-assay coefficient of variation was <10% for all assays.

Hormone levels were measured during the diestrous stage of the estrous cycle as determined by monitoring vaginal epithelial cell smears [29]. In brief, proestrus is characterized by distinctively round, nucleated epithelial cells and the complete absence of leukocytes; estrus is characterized by masses of clustered, cornified squamous epithelial cells; metestrus is characterized by a mixture of leukocytes and epithelial cells; and diestrus is characterized by large numbers of leukocytes [30].

### Statistical analysis

All experimental data were analyzed by a Student *t*-test or one-way ANOVA, using SPSS 17.0 software (SPSS, Chicago, IL, USA). *P*<0.05 was taken to indicate statistical significance. All values are presented as means ± SEM.

## Results

### Screening a porcine BAC library

**Figure 1:**
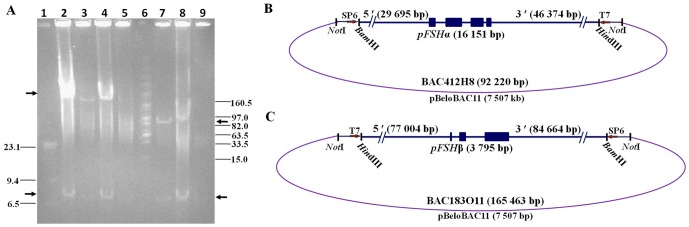
Pulsed-field gel electrophoresis (PFGE) and schematic representation of BAC clones spanning *pFSHα* and *pFSHβ*. (A) Lane 1: λ-*Hin*dIII marker; lanes 2–5: *Not*I-digested BAC183O11 DNA at different concentrations (3 µg, 0.2 µg, 1 µg, 0.1 µg); lane 6: MidRange II PFG marker; lanes 7–9: *Not*I-digested BAC412H8 DNA at different concentrations (0.2 µg, 0.8 µg, 0 µg); upper arrows, linear BAC bands; lower arrows, vector bands. (B–C) BAC clones were mapped by a combination of restriction endonuclease digestion, PFGE, direct DNA sequencing and alignment with the published gene sequence. Solid boxes represent gene exons and dashed lines represent two flanking sequences. The positions of the *Not*I restriction sites are indicated by the vertical bars.

BAC412H8 and BAC183O11 spanning the entire porcine pituitary glycoprotein hormone common α-subunit gene (GenBank: D00768) [31] and FSH β-subunit gene (GenBank: D00621) [32], respectively, were isolated from a porcine BAC library. After *Not*I digestion and pulsed-field gel electrophoresis, the inserts of BAC412H8 and BAC183O11 were found to be approximately 92 kb and 165 kb, respectively (Figure 1A). Sequencing demonstrated that BAC412H8 spanned 92 220 bp and contained 29 695 bp of 5′-flanking sequences and 46 374 bp of 3′-flanking sequences (Figure 1B). BAC183O11 contained 165 463 bp, flanked by 77 004 bp of upstream DNA and 84 664 bp of downstream DNA (Figure 1C). BLAST analysis indicated that both BAC clones did not contain any other genes.

### Generation and identification of BAC TG mice

**Figure 2:**
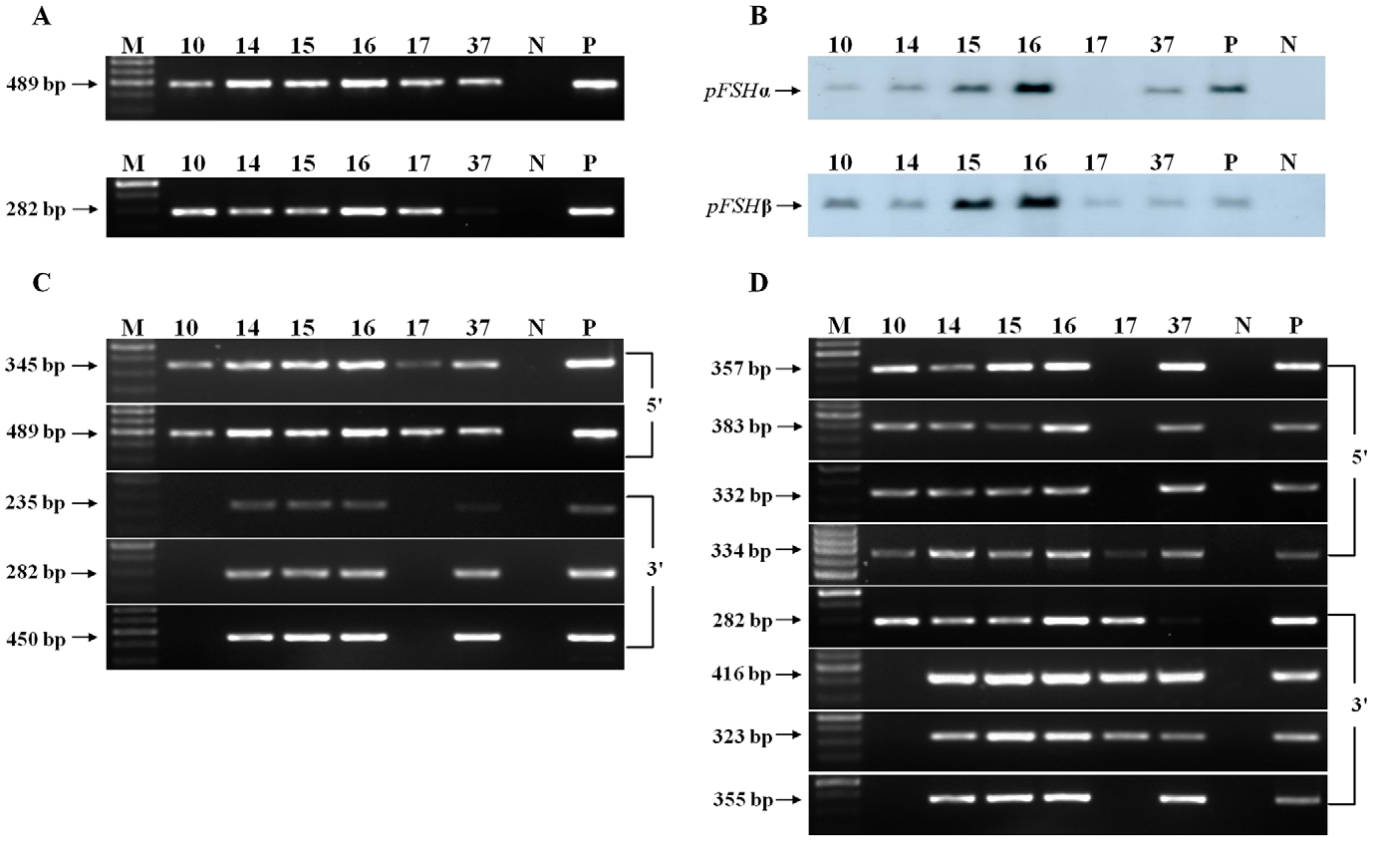
Identification of TG mice and assessment of the integrity of BAC transgenes in TG mice. (A) PCR for detecting TG mice. Two pairs of primers were used to amplify the 489 bp and 282 bp inserts. M, 100 bp DNA ladder; 10–37, TG founders; N, genomic DNA from WT mouse as a negative control; P, circular BAC DNA as a positive control. (B) Southern blot analysis of integrated pFSH transgenes. Purified genomic DNA (10 µg) from each TG founder was digested by *Bam*HI overnight at 37°C and analyzed by Southern blotting using probes specific for *pFSHα* and *pFSHβ*. 10–37, TG founders; P, porcine genomic DNA as positive control; N, genomic DNA from a WT mouse as a negative control. Band intensity was quantified by densitometry using Quantity-One software (Bio-Rad). (C–D) The two ends of both the BAC412H8 and BAC183O11 transgenes were amplified separately from TG mouse genomic DNA using 5′- or 3′-specific primers.

We created BAC TG mice by co-injecting two linear *Not*I fragments from BAC412H8 and BAC183O11 DNA into the pronuclei of zygotes from KM mice. Founder TG mice were first identified by PCR (Figure 2A) and then confirmed by Southern blotting (Figure 2B) of tail DNA. Of 40 pups born, six animals were positive for both *pFSHα* and *pFSHβ* integration by both methods. Transgene copy numbers were estimated by Southern blot and quantitative real-time PCR. The results indicated that in all lines there were 1–4 copies of both transgene inserts per diploid genome (Table 1).

**Table 1:** Summary of BAC TG lines.

Transgenic line	Insert gene	Copy number			BAC intactness	Specific expression
		F0	F1	F2		
10 *♂*	α	1	1	1	No	UD
	β	1	1	1	No	Yes
14 *♂*	α	1	1	1	Yes	Yes
	β	2	2	1–2	Yes	Yes
15 *♂*	α	2–3	2–3	2–3	Yes	Yes
	β	1–2	2	2	Yes	Yes
16 *♂*	α	3–4	3–4	3–4	Yes	Yes
	β	2–3	2–3	2–3	Yes	Yes
17*♀*	α	1	1	1	No	UD
	β	1	1	1	No	Yes
37 *♂*	α	1–2	1–2	1–2	Yes	Yes
	β	1	1	1	Yes	Yes

Each TG line was derived from one independent founder mouse. UD, Undetectable.

The integrity of the BAC transgenes in the TG lines was assessed by amplifying the 5′- and 3′-flanking regions of the linear BAC from tail DNA. The BAC DNA from four founders was intact whereas the remaining two TG founders carried deleted sections of the BAC transgenes (Figure 2C–D). All six founders were crossed to WT mice; transmission was observed in all cases, although line 10 transmitted the transgene only to male mice, suggesting that the transgenes had integrated into the Y chromosome. The F1 and F2 offspring of all six founders were analyzed by PCR and Southern blot for the presence of the integrated transgenes (Table 1). As shown in Table 2, all TG lines, with the exception of line 10, transmitted the two genes in Mendelian proportions, indicating that the two transgenes are present as co-integrants at a single (or two closely linked) chromosomal site(s).

**Table 2:** Transmission of *pFSHα* and *pFSHβ* BAC transgenes in TG×WT crosses.

Generation	Gender	Transgenic pups/total pups (ratio)
F1	*♀*	61/172 (35.47%)
	*♂*	97/183 (53.01%)
F2	*♀*	131/270 (48.52%)
	*♂*	152/279 (54.48%)
F3	*♀*	43/90 (47.78%)
	*♂*	52/101 (51.49%)
F4	*♀*	57/104 (54.81%)
	*♂*	48/111 (43.24%)

No difference in transgene transmission frequencies via the female versus male germlines were observed (with the exception of line 10 where the transgene insertion site appears to be within the Y chromosome).

### Expression analysis of *pFSHα* and *pFSHβ*

**Figure 3:**
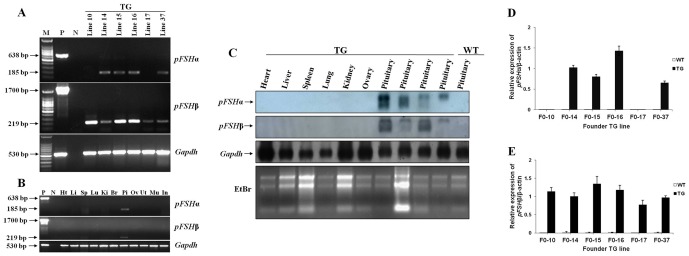
Tissue expression of *pFSHα* and *pFSHβ* in adult TG mice. (A–B) RT-PCR analysis of RNA extracted from the pituitary and other tissues from mice of the six different TG lines. *Gapdh* mRNA was used as a control. Ht, heart; Li, liver; Sp, spleen; Lu, lung; Ki, kidney; Br, brain; Pi, pituitary; Ov, ovary; Ut, uterus; Mu, muscle; In, intestine; M, 100 bp DNA ladder; P, upper and middle lanes, expression vector as a positive control and, lower lane, pituitary of a WT mouse; N, upper and middle lanes, pituitary of a WT mouse as a negative control and, lower lane, double-distilled water. (C) Northern blot analysis of transgene expression in adult tissues. Northern hybridization with the indicated cDNA probes was carried out using total RNA (20 µg) isolated from different tissues of TG and WT mice. Ethidium bromide (EtBr) staining of 28S, 18S, and 5S rRNA served as a control for RNA quality. Pituitary samples from four TG animals were detected. (D–E) *pFSHα* and *pFSHβ* mRNA levels in the pituitaries of TG and WT mice were analyzed using quantitative real-time PCR with specific primers and are expressed relative to β-actin (internal control). Data were combined from three independent experiments; bars represent means ± SEM (*n* = 7–8 mice per group).

To investigate whether the major regulatory elements for *pFSHα* and *pFSHβ* expression in the pituitary are present in the BAC412H8 and BAC183O11 transgenes, RNA isolated from pituitary and other tissues of F1 mice from different lines was analyzed by RT-PCR (Figure 3A–B). mRNAs for both subunits were detected in the pituitaries of four lines, whereas in lines 10 and 17 only β-subunit mRNA was detected in the pituitary. This may reflect the fact that the 3′ end of the linear BAC412H8 transgene was deleted in both lines 10 and 17. In all expressing lines, the transgenes were expressed specifically in the pituitary (Table 1).

We further analyzed mRNA from TG lines 14 and 37 for *pFSHα* and *pFSHβ* expression in different tissues by northern blotting (Figure 3C) employing cDNA probes specific for *pFSHα* and *pFSHβ*; a *Gapdh* cDNA probe was used as an internal control for RNA loading. Expression was only observed in pituitary; no ectopic expression was observed in the other tissues examined. These results indicate that the major regulatory elements (promoter and enhancer sequences) required for tissue-specific expression of *pFSHα* and *pFSHβ* are present within the BAC412H8 and BAC183O11 transgenes, respectively.

To evaluate the relative levels of expression of *pFSHα* and *pFSHβ* in TG mice, pituitaries were collected from F1 TG mice and from WT littermates; mRNA was quantified using quantitative real-time PCR (Figure 3D–E). No transcripts were detected in pituitaries of WT mice. Pituitaries from TG mice showed high expression of *pFSHα* and *pFSHβ* mRNA, although α-subunit mRNA was not detected in lines 10 and 17. There was no overt correlation between pituitary mRNA levels and transgene copy number.

To verify the presence of circulating pFSH, serum samples from adult TG mice and from age-matched WT littermates were analyzed by ELISA using a polyclonal antibody against pFSH that does not cross-react with endogenous mouse FSH. pFSH was readily detected in TG serum and levels of pFSH in independent TG lines ranged from 6.36 to 19.83 IU/L, within the physiological range. Mean levels of pFSH in serum of mouse lines 14 and 37 was 11.72±0.67 IU/L (Table 3).

**Table 3:** Serum levels of FSH in TG and WT female mice.

Genotype	Porcine FSH (IU/L)	Mouse FSH (IU/L)
WT	UD (n = 8)	3.13±0.39 (n = 20)
TG	11.72±0.67 (n = 12)	4.84±1.04** (n = 18)

** *P*<0.01. UD, Undetectable. All values represent means ± SEM. n = number of animals. The results of the analysis of each of the two separate lines were found to be comparable to that of the combined analysis.

### Effect of pFSH overexpression on female fertility

**Figure 4:**
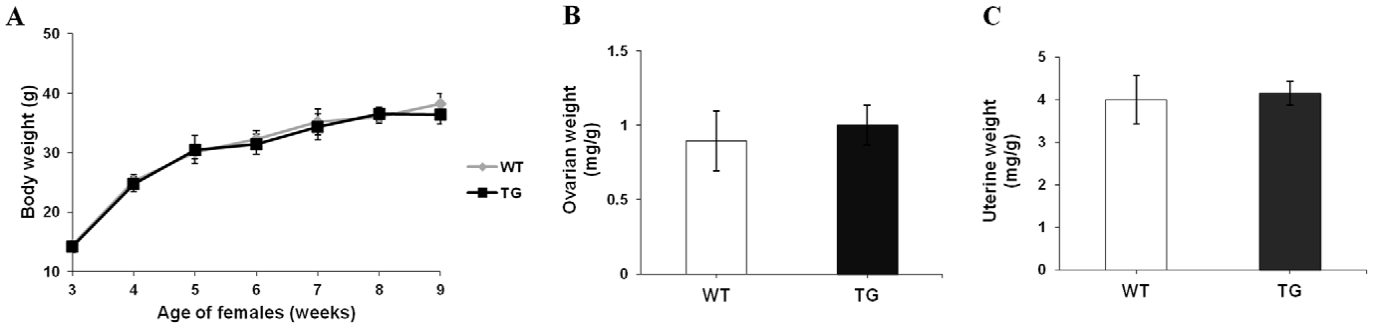
Effects of *pFSHα* and *pFSHβ* expression on body and reproductive organ weights. (A) Growth curves for TG and WT females from birth to 9 weeks of age. (B–C) Ovary- and uterus-to-body weight ratios (mg/g) for TG (*n* = 12) and age-matched WT (*n* = 11) females (19–21 weeks of age). No effect was detected. The results of the analysis of each of the two separate lines were found to be comparable to that of the combined analysis.

**Figure 5:**
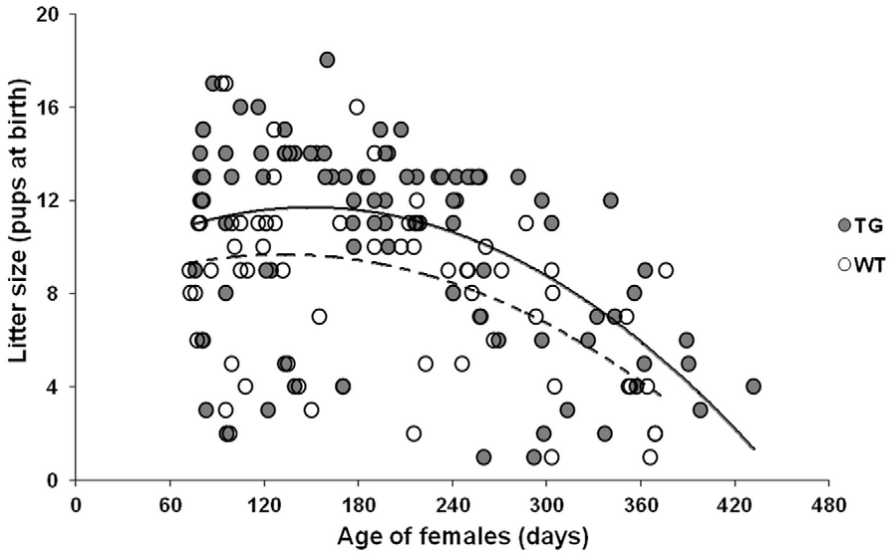
Reproductive performance of TG females. Fertility data on liveborn pups from TG (*n* = 15) and WT (*n* = 15) female mice up to 450 days of age; the number of litters per mouse was approximately six. Total pups per litter plotted against age of female on the day of birth showing comparable breeding curves for the TG (solid line) and WT (dashed line) females. The results of the analysis of each of the two separate lines were found to be comparable to that of the combined analysis.

Expression of pFSH had no detectable effect on body weight or on the steroid hormone-responsive sizes of the ovary or uterus (Figure 4). By contrast, the expression of pFSH had an obvious effect on female fecundity. From 9–10 weeks of age, WT and TG females of lines 14 and 37 were housed with fertile WT mates over their breeding lifetime. Female mating was confirmed by copulatory plugs and litter size was determined on the day of birth. Breeding curves for both TG and WT females showed that litter sizes first increased (≤180 days of age) and then decreased until the females were infertile. In both TG and WT females the largest litter sizes were observed at 180 days of age (Figure 5). However, TG females had significantly larger litter sizes than the WT controls (11.5±4.0 vs 9.3±3.9 pups per litter, *P*<0.001); there were no significant differences between TG and WT mice in the delay between successive litters (*P* = 0.67). Furthermore, TG female mice had significantly larger total number of pups than their WT littermates (69.7±3.5, n = 15 vs 56.0±3.3, n = 15; P<0.001) within the examination period. All pubs were viable and survived, but most of offspring produced were euthanized shortly after counting them and only a fraction of pups were selected randomly and maintained for histological and hormonal analyses.

### Effect of pFSH overexpression on corpora lutea number

**Figure 6:**
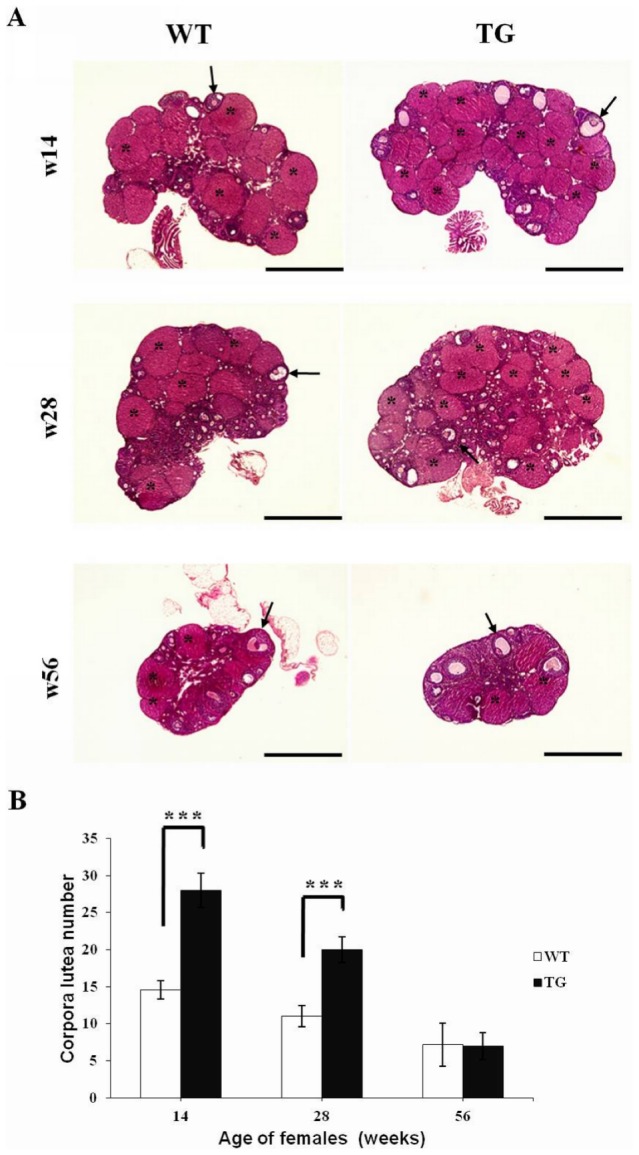
Effects of *pFSHα* and *pFSHβ* expression on the number of ovarian corpora lutea. (A) Ovarian sections (5 µm) from WT and TG females at 14, 28, and 56 weeks of age were stained with H&E. Antral follicles, arrows; corpora lutea, asterisks; scale bars, 2.0 mm. (B) Total number of ovarian corpora lutea during the diestrous stage of the estrous cycle in TG and WT females at 14, 28, and 56 weeks of age; *n* = 6–8 mice per genotype; ***P<0.001. The results of the analysis of each of the two separate lines were found to be comparable to that of the combined analysis.

To determine if the improved fecundity in TG females was due to an increased number of ovulatory follicles, we compared the number of ovarian corpora lutea in TG and WT females of lines 14 and 37. Ovarian histology revealed that primordial, primary, preantral, and antral follicles and corpora lutea were present at diestrus in 14- to 56-week-old TG and WT females (Figure 6A). The total number of ovarian corpora lutea was significantly increased in TG mice as compared with WT mice at 14 (28.0±2.3, *n* = 8 vs 14.6±1.3, *n* = 6; *P*<0.001) and 28 (20.0±1.7, *n* = 8 vs 11.0±1.4, *n* = 8; *P*<0.001) weeks of age (Figure 6B), suggesting that the increase in litter size was due to enhanced ovulation. There was no significant difference in the number of corpora lutea in 56-week-old TG and WT mice (7.0±1.8, *n* = 7 vs 7.2±2.9, *n* = 8; *P* = 0.86). In addition, no pathological abnormalities were observed in the overall histology of TG versus control mice (Figure S1).

### Serum FSH, LH, estradiol, and testosterone concentrations in TG and WT mice

**Figure 7:**
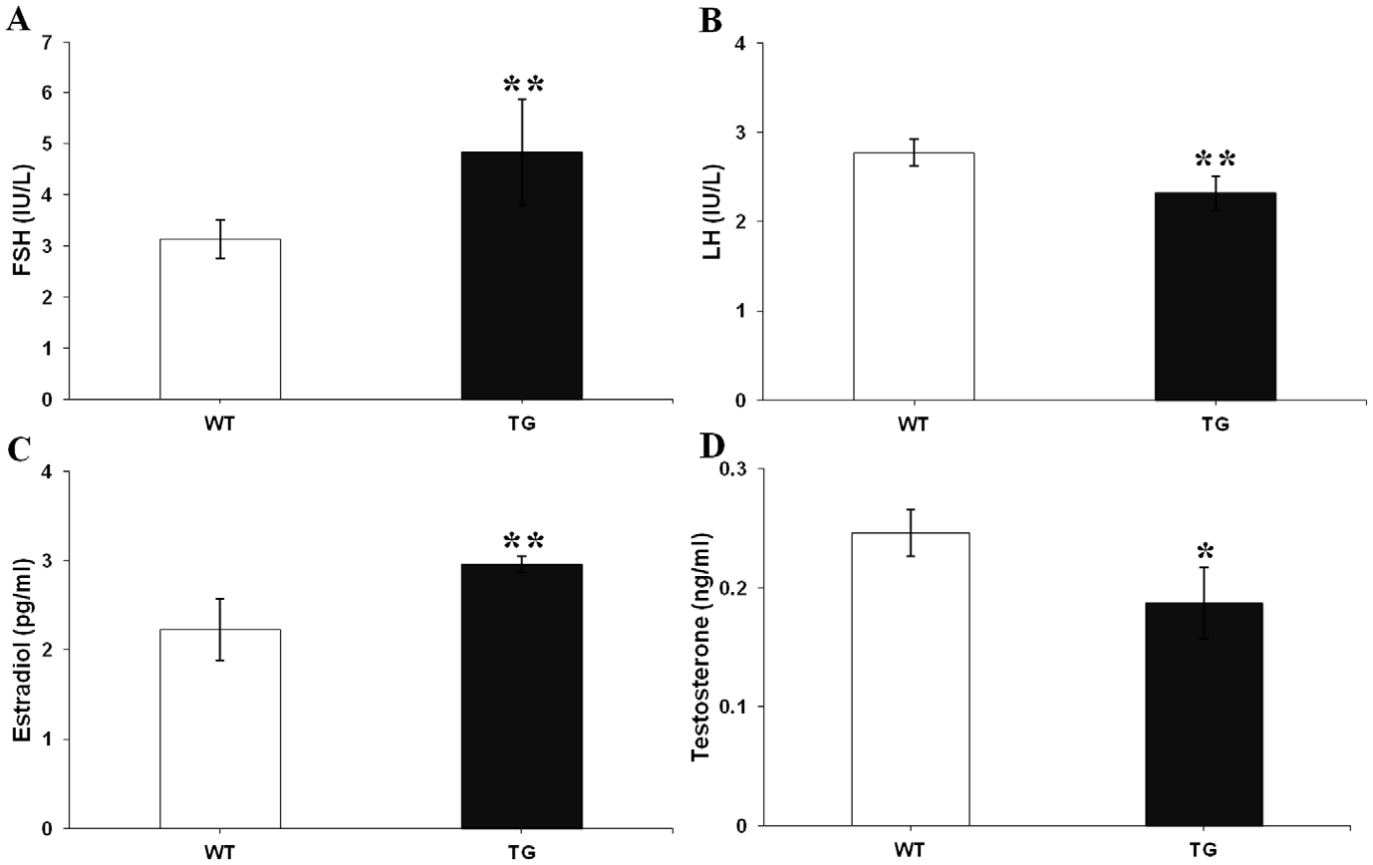
Serum FSH, LH, estradiol, and testosterone levels in TG and WT mice. Blood was collected from TG and WT mice at 19–21 weeks of age during the diestrous stage of the estrous cycle for measurement of mouse FSH (A), LH (B), estradiol (C), and testosterone (D) (*n* = 18–20 mice per genotype). Data were combined from three independent experiments; bars represent means ± SEM; *P<0.05; **P<0.01. The results of the analysis of each of the two separate lines were found to be comparable to that of the combined analysis.

**Figure 8:**
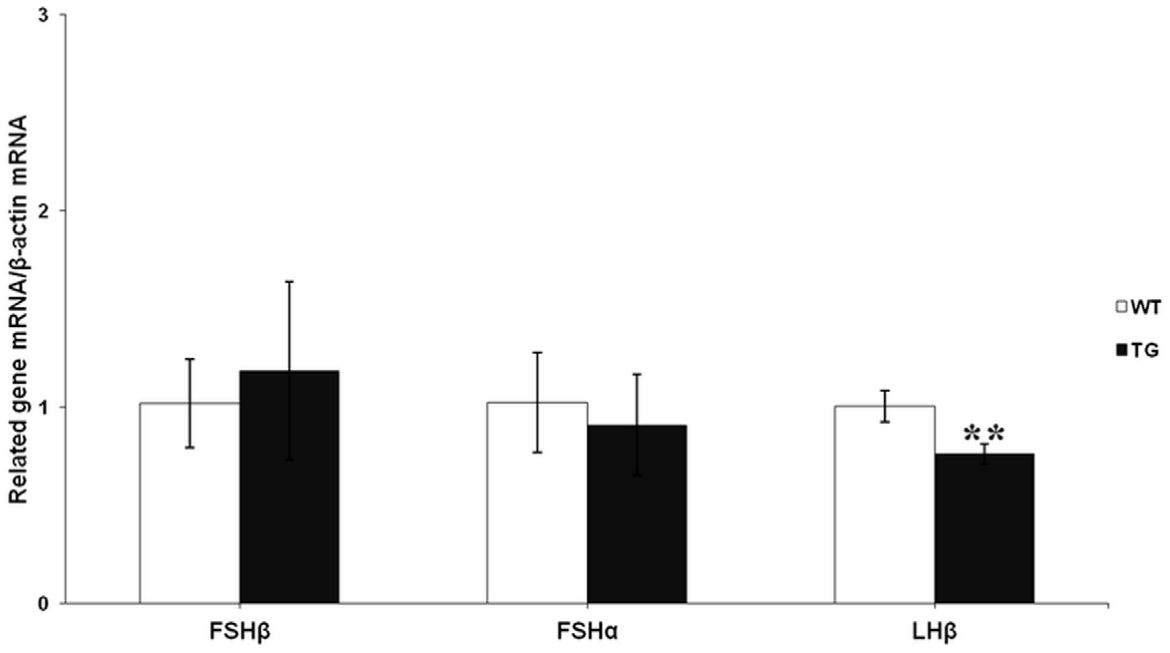
Pituitary *FSHα*, *FSHβ* and *LHβ* mRNA expression in TG and WT mice. Mouse *FSHα*, *FSHβ* and *LHβ* mRNA were analyzed using quantitative real-time PCR with specific primers and were expressed relative to a β-actin (internal control) (*n* = 7–8 mice per genotype). Data were combined from three independent experiments; bars represent means ± SEM; **P<0.01. The results of the analysis of each of the two separate lines were found to be comparable to that of the combined analysis.

Follicular growth, differentiation, atresia, and female fertility are regulated by hormones [33]. Therefore, the levels of FSH, LH, estradiol, and testosterone were compared in TG and control females of lines 14 and 37 during diestrus at 19–21 weeks of age (Figure 7). Serum levels of endogenous mouse FSH were significantly higher in TG mice as compared with WT littermates (4.84±1.04 IU/L, *n* = 18 vs 3.13±0.39 IU/L, *n* = 20; *P*<0.01) (Figure 7A). Serum levels of LH were significantly lower in TG mice as compared with WT littermates (2.32±0.19 IU/L, *n* = 18 vs 2.77±0.15 IU/L, *n* = 20; *P*<0.01) (Figure 7B); meanwhile we observed that TG pituitaries had a significantly lower expression of LHβ mRNA as compared to WT pituitaries (Figure 8). Estradiol levels were significantly higher in TG mice as compared with WT littermates (2.96±0.09 pg/ml, *n* = 18 vs 2.23±0.34 pg/ml, *n* = 20; *P*<0.01) (Figure 7C). Testosterone levels were significantly lower in TG mice as compared with WT littermates (0.19±0.03 ng/ml, *n* = 18 vs 0.25±0.02 ng/ml, *n* = 20; *P*<0.05) (Figure 7D). In addition, the alteration of circulating endogenous hormone did not affect peripheral blood cell levels in TG mice as compared with WT littermates (Table S2).

## Discussion

BAC transgenesis represents a major breakthrough in the functional generation of TG animals [34] and has been applied to devise new animal models of human genetic diseases, to study gene function during development, and to produce recombinant proteins in the mammary glands of transgenic animals [35], [36], [37], [38], [39], [40]. Substantial progress has been made in the generation of TG mouse lines that carry large fragments of genomic DNA contained in BACs [20]. We report, for the first time, the incorporation into the mouse genome of complete BAC DNAs encompassing the two pFSH-subunit genes, allowing us to investigate the phenotypic expression of an entire gene in native environment. The efficiency of transgenesis with BACs in our study was about 15%, which is comparable with that of standard plasmid constructs (5–20% of newborn animals). All six TG founders carried a low number of copies (1–4) of each transgene, suggesting that only a few BAC DNA molecules were introduced into each fertilized egg, although the integration of up to 76 copies of a BAC transgene has been reported previously [41]. All TG lines transmitted the two genes to the F4 generation, suggesting that the two transgenes are stably incorporated into the mouse genome.

Our BAC TG mice have shown that pFSH mRNA can be expressed successfully under the pFSH α- and β-subunit gene promoters in pituitary gonadotroph cells, suggesting that the regulatory regions of the *pFSHα* and *pFSHβ* transgenes comprise almost all the relevant binding sites for *trans*-acting factors, and that the cognate mouse DNA-binding proteins can form transcriptional complexes with these regions to direct accurate tissue-specific expression. Kumar and colleagues previously reported that a 10 kb human FSH β-subunit genomic DNA clone containing 4 kb of 5′-flanking sequences and 2 kb of 3′-flanking sequences also conferred gonadotroph-specific expression and appropriate hormonal regulation in the pituitaries of TG mice [10], [11], [12], [42], but these studies only addressed the expression of the human FSH β-subunit and lacked distant regulatory sequences. Levels of circulating pFSH (∼11 IU/L) in our TG mice did not exceed physiological levels (e.g. fertile KM mice, 3–20 IU/L). Interestingly, breeding data revealed that, until 52 weeks of age, our TG females with elevated pFSH levels produced increased litter sizes relative to WT littermates, but did not appear to lead to early depletion of the follicle reserve. We predict that more antral follicles rescued from apoptosis are selected as the dominant follicle during cyclic recruitment as a result of elevated circulating FSH. By contrast with studies in two other mouse models: One is that TG female mice, expressing supraphysiological human FSH levels (362,000 IU/L) under the control of the mouse metallothionein-1 (MT) promoter [27], [43], were infertile with abnormal gonadal hyperplasia such as hemorrhagic or cystic ovaries and enlarged uteri; the other is that expression of human FSH (2.5–10 IU/L), driven by the rat insulin II gene promoter, had a marked effect on female mouse fertility that was both biphasic and age-dependent [44], [45], [46]. It is because of ectopic overexpression of human FSH independently of GnRH regulation, resulting in progressively rising FSH levels, with increasing embryo–fetal resorption and parturition failure as the TG animal age. Our results demonstrate that joint expression of both pFSH subunits in TG mice within the physiological range does not disrupt normal female reproductive function. The reasons for differences in the results of our model and previous models are unclear, but it is likely that they stem from differences in the species used for FSH, the specific tissues targeted by FSH and/or the level of overexpression.

To investigate further the mechanisms underlying the improved reproductive ability observed in pFSH-overexpressing TG females, ovarian histology was assessed at 14, 28, and 56 weeks of age. Previous data have indicated that FSHβ deletion [5] and FSHβ overexpression [27] significantly affect ovarian development and function. Specifically, FSHβ knockout female mice were infertile due to a block in folliculogenesis before antral follicle formation [5] whereas TG female mice overexpressing human FSHβ under the control of a mouse MT promoter were also infertile due to disrupting ovarian folliculogenesis [27]. In our study the TG female mice were fertile and the total numbers of ovarian corpora lutea were significantly increased compared to WT littermates at 14 and 28 weeks of age. The increased litter sizes and number of corpora lutea suggest that elevated FSH activity results in enhanced ovulation. This suggestion is consistent with previous findings of increased ovulation in TG mice with elevated pituitary-derived FSH expression [47], and elevated ovarian FSH sensitivity in sheep with a Booroola mutation that affects the bone morphogenic protein pathway and drives increased ovulation rates and litter sizes [48]. These findings suggest that elevated FSH activity can enhance follicle survival before ovulation, particularly for small antral follicles.

Hormonal regulation of follicular growth, differentiation, and apoptosis is crucial for normal ovarian development and function [33], [49]. Our TG female mice have significantly lower levels of LH, and testosterone, significantly higher levels of estradiol and FSH levels compared with WT littermates. Endogenous FSH levels in TG mice were elevated primarily due to pFSH expression, analogous to induction of ovulation with recombinant human FSH in women. The increased levels of estradiol observed in our TG mice might reflect an enhanced capacity for aromatization of androgens to estrogens due to elevated FSH bioactivity; in contrast, testosterone levels were decreased as a result of low LH levels in TG mice, and/or the lower testosterone levels may be due to aromatization of androgens to estrogens, in accordance with the two cell–two gonadotropin theory [50]. These changes led to a depressed androgen/estrogen ratio, therefore our TG female mice did not show reproductive abnormalities (e.g. polycystic ovarian syndrome). FSH and LH share a common α subunit which is thought to be secreted in excess relative to the β subunit [3]; thus, the amount of FSH and LH principally depends on the synthesis of specific β subunit. In fact, we observed a reduction of LHβ mRNA levels in our TG mice. We presume that overexpression of pFSH may affect the synthesis of LHβ, but the exact mechanism for this is unclear.

In conclusion, we have successfully generated a novel gain-of-function TG mouse in which extensive 5′- and 3′-flanking sequences confer tissue-specific expression of both *pFSHα* and *pFSHβ*. Moreover, our findings indicate that rising basal levels of FSH in pituitary gonadotroph cells increase ovarian ovulation rates and thereby increase litter sizes, but without affecting embryo quality or uterine function, suggesting the importance of pituitary targeted expression of pFSH in reproductive physiology. We propose that increased FSH of Chinese Erhualian pig, within the physiological range, does not induce reproductive defects and may, in fact, improve female reproductive performance. In the future deletion analyses within the 5′- and 3′-flanking sequences of the pFSH α- and β-subunit genes in transgenic mice should provide further insights into the underlying molecular mechanisms of regulation of pFSH α- and β-subunit genes.

## Supporting Information

Figure S1 **General histology of WT and TG female mice.** Brain, pituitary, ovary and uterus sections (5 µm) from WT and TG female mice at 10 weeks of age were stained with H&E. No differences were seen in the overall histology of TG versus control mice. Bar indicates 20 µm.
(TIF)

Table S1 Primers used for the identification of BAC TG mice.
(DOC)

Table S2 Peripheral blood cell levels in WT and TG mice.
(DOC)
