# Supplementary figures and images for: Pituitary-Specific Overexpression of Porcine Follicle-Stimulating Hormone Leads to Improvement of Female Fecundity in BAC Transgenic Mice
Source: PLoS One. 2012 Jul 31;7(7):e42335. doi: 10.1371/journal.pone.0042335 (PMC3409198; doi:10.1371/journal.pone.0042335)

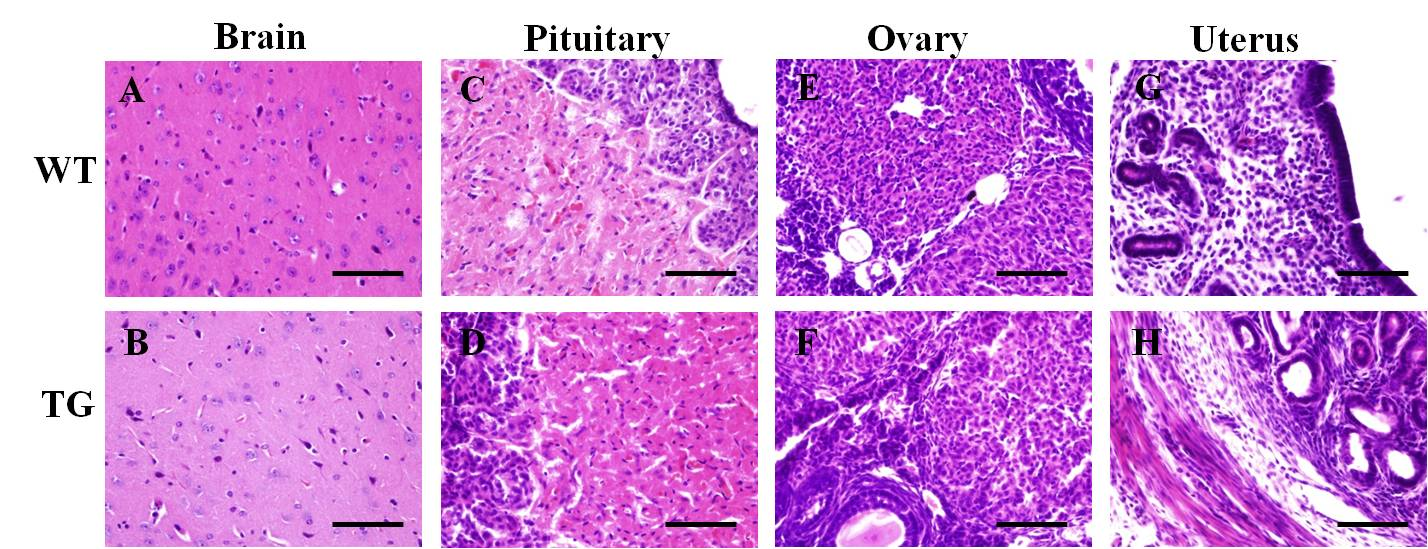

Supplement: Figure S1 — General histology of WT and TG female mice. Brain, pituitary, ovary and uterus sections (5 µm) from WT and TG female mice at 10 weeks of age were stained with H&E. No differences were seen in the overall histology of TG versus control mice. Bar indicates 20 µm. (TIF) [file pone.0042335.s001.tif]
