# Supplementary material for: Pituitary-Specific Overexpression of Porcine Follicle-Stimulating Hormone Leads to Improvement of Female Fecundity in BAC Transgenic Mice
Source: PLoS One. 2012 Jul 31;7(7):e42335. doi: 10.1371/journal.pone.0042335 (PMC3409198; doi:10.1371/journal.pone.0042335)
Supplement: Table S1 — Primers used for the identification of BAC TG mice. (DOC) [file pone.0042335.s002.doc]

**Table S1**Primers used for the identification of BAC TG mice.

|  | **Primer name** | **Sense primer (5′→3′)** | **Antisense primer (5′→3′)** |
| --- | --- | --- | --- |
| PCR | *pFSHα*-BAC(15kb) | TGCCAAGCCGTAATAGGAACT | TCTAGGGGTCAAATCAGAGCT |
|  | *pFSHα*-BAC(30kb) | AGCCTGAGGCACTTAGTAGGT | CAAGGTCAGCCTATGAAACAA |
|  | *pFSHα*-BAC(45kb) | GCCTTGTTGATGACTGCTGA | TTTCCAGCAGAACCAGCTTT |
|  | *pFSHα*-BAC(75kb) | GCCATGTAGATGTCTAAGGAA | AGTATGAGA ACCTCTAGGCTC |
|  | *pFSHα*-BAC(90kb) | AAGCGTTTTGTACCATGTGAC | TGACGGGCATTATCTGATACT |
|  | *pFSHβ*-BAC(5kb) | CTTCCCAGATTGTCCTCATTT | ATGCATGAAGCCTTGATACCT |
|  | *pFSHβ*-BAC(25kb) | GGGAGAGCATAACAAGGTC | TGGAGCTCACATTGAACACT |
|  | *pFSHβ*-BAC(45kb) | CCTCCAGGACTATGCGACA | TGTGCCACTGCCACCTACTG |
|  | *pFSHβ*-BAC(70kb) | GAACACTGAGGGAGGAGCTG | GGGGAACTCCAGCACTGATA |
|  | *pFSHβ*-BAC(80kb) | CTACAAGAGCAGGGCCAGTC | CCTGCCTGAGAGAGATGTCC |
|  | *pFSHβ*-BAC(105kb) | GACCACCACTGAAGCATCTCT | TATCCGTGTGCTTCAGGCTAT |
|  | *pFSHβ*-BAC(130kb) | TTCTCCTGGGAACCTATCAA | ACCACCCTCTCGTTTAAGC |
|  | *pFSHβ*-BAC(155kb) | GGCTAAGTTCTGGATCACC | GCCAGTCTCACCTTATGCAA |
| Real-time PCR (DNA) | *pFSHα* | AACTGCCTCCTCCAGAGATGTG | TCCCTGGTAGCCACTAAAAGAATG |
|  | *pFSHβ* | ACACGCAGTTTATGGCAATGC | CATTTGGCTCAGCAAGTTAAGAAC |
|  | *Fabpi* | GACTGCTGGTCCTCCTACAGGAT | ATTTGCACCCAACCAATGGA |
| Real-time PCR (RNA) | *pFSHα* | CATCACCTCGGAAGCCACAT | CAGTGGCATTCGGTGTGGTT |
|  | *pFSHβ* | CACGTGGTGTGCTGGCTATT | CGGTCTCGTACACCAGCTCC |
|  | *β*-actin | TTCTACAATGAGCTGCGTGTGG | GGTGTTGAAGGTCTCAAACATGAT |
|  | Mouse-*FSHα* | CAGGGTTGCCCAGAATGTAAA | TGGCAGGAGTGGGATATGC |
|  | Mouse-*FSHβ* | GTGTGCGGGCTACTGCTACA | ACAGCCAGGCAATCTTACGG |
|  | Mouse-*LHβ* | CTGGCCGCAGAGAATGAGTT | TGAGGCACAGGAGGCAAAG |

Common PCR primers were designed by Oligo 6.0 software (Molecular Biology Insights, Inc.) and real-time PCR primers were designed by Primer Express 3.0 software (Applied Biosystems).
