# Supplementary material for: Pituitary-Specific Overexpression of Porcine Follicle-Stimulating Hormone Leads to Improvement of Female Fecundity in BAC Transgenic Mice
Source: PLoS One. 2012 Jul 31;7(7):e42335. doi: 10.1371/journal.pone.0042335 (PMC3409198; doi:10.1371/journal.pone.0042335)
Supplement: Table S2 — Peripheral blood cell levels in WT and TG mice. (DOC) [file pone.0042335.s003.doc]

**Table S2 Peripheral blood cell levels in WT and TG mice.**

| **Peripheral blood cell** | **WT (n = 16)** | **TG (n = 12)** |
| --- | --- | --- |
| WBC (G/L) | 4.3±0.97 | 4.59±1.22 |
| RBC (T/L) | 12.88±0.89 | 12.35±0.98 |
| HGB (g/L) | 165.7±12.79 | 159.5±19.46 |
| HCT (%) | 50.29±3.17 | 48.75±3.98 |
| MCV (fL) | 38.93±1.45 | 39.3±1.08 |
| MCH (pg) | 12.81±0.58 | 12.83±0.78 |
| MCHC (g/L) | 329.4±11.02 | 326.38±14.69 |
| PLT (G/L) | 312.4±57.70 | 309.25±76.92 |

All values represent means ± SEM. n = number of animals.
